# Elements of effective palliative care models: a rapid review

**DOI:** 10.1186/1472-6963-14-136

**Published:** 2014-03-26

**Authors:** Tim Luckett, Jane Phillips, Meera Agar, Claudia Virdun, Anna Green, Patricia M Davidson

**Affiliations:** 1 Improving Palliative Care through Clinical Trials (ImPaCCT), Sydney, NSW, Australia; 2 University of Technology Sydney (UTS), Faculty of Health, Building 10, Level 7, 235-253 Jones St, Ultimo, NSW 2007, Australia; 3 South Western Sydney Clinical School, University of New South Wales (UNSW), Sydney, NSW, Australia; 4 The Cunningham Centre for Palliative Care Sydney, Sacred Heart Hospice, Sydney, NSW, Australia; 5 School of Nursing, The University of Notre Dame Australia, Sydney, NSW, Australia; 6 HammondCare, Sydney, NSW, Australia; 7 The Ingham Institute for Applied Medical Research, Sydney, NSW, Australia; 8 School of Nursing, Johns Hopkins University, Baltimore, MD, USA

**Keywords:** Palliative care, Models of care, Rapid review, Case management, Home based care, Nursing homes, Specialist palliative care, Acute care, Emergency departments

## Abstract

**Background:**

Population ageing, changes to the profiles of life-limiting illnesses and evolving societal attitudes prompt a critical evaluation of models of palliative care. We set out to identify evidence-based models of palliative care to inform policy reform in Australia.

**Method:**

A rapid review of electronic databases and the grey literature was undertaken over an eight week period in April-June 2012. We included policy documents and comparative studies from countries within the Organisation for Economic Co-operation and Development (OECD) published in English since 2001. Meta-analysis was planned where >1 study met criteria; otherwise, synthesis was narrative using methods described by Popay et al. (2006).

**Results:**

Of 1,959 peer-reviewed articles, 23 reported systematic reviews, 9 additional RCTs and 34 non-randomised comparative studies. Variation in the content of models, contexts in which these were implemented and lack of detailed reporting meant that elements of models constituted a more meaningful unit of analysis than models themselves. Case management was the element most consistently reported in models for which comparative studies provided evidence for effectiveness. Essential attributes of population-based palliative care models identified by policy and addressed by more than one element were communication and coordination between providers (including primary care), skill enhancement, and capacity to respond rapidly to individuals’ changing needs and preferences over time.

**Conclusion:**

Models of palliative care should integrate specialist expertise with primary and community care services and enable transitions across settings, including residential aged care. The increasing complexity of care needs, services, interventions and contextual drivers warrants future research aimed at elucidating the interactions between different components and the roles played by patient, provider and health system factors. The findings of this review are limited by its rapid methodology and focus on model elements relevant to Australia’s health system.

## Background

Access to appropriate care and support at the end of life is recognised by many as a basic human right [1]. However, ongoing changes in disease and society demand rethinking who should properly receive such care, and how, where and from whom they should receive it. The traditional focus on specialist palliative care (SPC) teams caring for people with cancer in a hospice or community setting has been expanded to include a wide range of life-limiting disease groups and care settings [2]. Changes in living and social circumstances mean that current generations can no longer expect the informal caregiving taken for granted by their forbearers, forcing people to look to formalised healthcare and social services. At the same time, individualistic, consumerist attitudes mean that people demand greater choice in determining and tailoring their healthcare, including the opportunity to be cared for and die in places of preference [3].

Advances in detection and treatment mean that diseases such as cancer and HIV that previously killed people quickly are now chronic conditions that confer an increasing burden of symptoms and functional decline over many years [4,5]. Medical advances have also contributed to population ageing, facing health systems with an increasing number and proportion of frail people with multiple conditions. Despite the best of intentions, this population is often ‘crisis managed’ within the acute care system rather than being adequately supported in the community for as long as possible [6]. In most countries, access to healthcare varies according to socioeconomic and geographic variables, mandating efforts to decrease health disparities [7,8]. Given the increasing number and changing profile of people with life-limiting illness, it is neither feasible nor desirable that SPC services provide care for everyone; rather, these services should be reserved for patients with the most complex palliative care needs [9]. A population approach to planning is therefore required that takes a ‘birds-eye view’ across the health system to inform the development of models of palliative care that integrate SPC with other services according to need [10,11].

The current study set out to inform Australian palliative care policy reform by identifying and synthesising:

1) recommendations for population based palliative care from international policy, and

2) the evidence for improvements on patient, family and health system outcomes available for different population-based models of palliative care from studies of any design comparing one model with another or models to usual care.

## Methods

A rapid review of the palliative care literature was undertaken over an eight week period in April 2012. Rapid review methodology utilises similar processes to a full systematic review but generates a more timely synthesis of the evidence by limiting scope (e.g. search terms and inclusion criteria ) and various aspects of synthesis (e.g. data extraction and bias assessment) [12,13]. In deciding which efficiency measures to introduce, researchers undertaking rapid reviews need to carefully weigh up advantages in time/resource saving against disadvantages in the form of decreased coverage and increased risk of bias; given an appropriate balance, a rapid review can generate adequate advice for the majority of clinical and policy decision when a pre-defined methodology is followed [12]. Decisions made in the current rapid review and associated limitations are summarised in relevant parts of the Methods and Discussion. A protocol was developed and discussed prior to commencement but was not made available publically.

### Eligibility criteria

Two kinds of document were deemed informative. First, we were interested to identify how various models of palliative care had been defined in the literature and which of these have been supported by evidence. We also sought international policy documents/reports with most applicability to Australia’s universal health care system and federal/state structure of funding. To be included, documents of both kinds needed to come from an Organisation for Economic Co-operation and Development (OECD) country and have been made publically available in English since 2001. We limited to more recent publications to maximise relevance to contemporary populations and healthcare contexts. Documents had to be concerned with facilitating the delivery of palliative care to people with progressive life-limiting illness in any setting.

Inclusion criteria relating to palliative care were based on the World Health Organisation (WHO) definition on the basis of being the most widely used internationally [14]. Models of care were defined as those providing a framework or system for the organisation of care for people with a progressive life-threatening illness and/or their family, carers or close friends [11]. Eligible care activities included those consistent with the aims of palliative care as defined by WHO (e.g. advance care planning and self-management) as well as meeting the care needs of the patient population (e.g. symptom management and care-giver support). In accordance with the WHO definition, inclusion criteria did not relate to the degree of training and/or experience of those providing care, but rather the nature of care provided. Indeed, as indicated in the Introduction, we were especially interested to identify evidence-based models of care involving generalist or primary palliative care providers as well as specialist services.

Studies were considered eligible for inclusion if they provided data on effectiveness and/or cost-effectiveness based on comparison either between two alternative models of palliative care or between a model of palliative care and usual care. Usual care was assumed to include routine community and hospital services other than SPC models (including private). Comparisons could be concurrent or historical. Studies providing level 1 and 2 evidence (systematic reviews and randomised controlled trials [RCTs]) were prioritised, with studies using other, less rigorous designs (e.g. multiple time series) being treated as secondary sources of information [15].

We were also interested to see which models of palliative care had been recommended by current international policy. Policy document were defined as any publically available statement of position, standards or recommendations officially put forward by a government. Eligible document types included reports by health services and peak bodies as well as peer-reviewed journal articles and books/book sections.

### Information sources

#### ***Electronic searches***

We searched Medline, AMED, CINAHL, the Cochrane Database of Systematic Reviews, Health Technology Assessment Database and CENTRAL from their earliest records. We also searched the grey literature via internet search engines (Google and Google Scholar), the online Australian palliative care knowledge network, CareSearch, and other relevant online clearinghouses (e.g. Americans for Better Care of the Dying). Deep web searching using Mednar was considered useful for the targeting of scientific material unavailable to search engines like Google [16]. Documents listed in CareSearch’s Review Collection relating to “Service/Systems Issues” (http://www.caresearch.com.au/caresearch/tabid/501/Default.aspx) were also reviewed for inclusion.

#### ***Other sources***

The reference lists of all included reviews were searched manually for further relevant articles.

### Search terms

Searches for literature reporting on palliative care were conducted simultaneously with those aimed at meeting secondary aims of identifying service planning tools and research on the palliative care needs of Australians (not reported in this paper). Database searches used Medical Subject Headings (MeSH) terms or equivalent as well as keywords relating to palliative and end of life care + service models (see Table 1 for an example). Search terms were based on those for PubMed developed by CareSearch.

**Table 1:** **Medline search terms used to identify relevant articles on palliative care models, service planning tools and palliative care needs of Australians in searches conducted on 4**^**th **^**April 2012**

	
1.	exp advance care planning/OR exp attitude to death/OR exp bereavement/OR exp terminal care/OR exp hospices/OR exp life support care/OR exp palliative care/OR exp terminally ill/OR death/OR palliate$.mp OR hospice$.mp OR terminal care.mp
2.	(dying.mp OR death.mp OR end of life.mp) AND (imminen$.mp OR nearing.mp OR last day$.mp OR last week.mp OR last hour$.mp OR final day$.mp OR final week.mp OR final hour$.mp OR critical pathway$.mp)
3.	1 OR 2
4.	exp delivery of health care/AND (exp models, theoretical/OR exp models, economic/)
5.	exp Community Health Planning/OR exp health care reform/OR exp decision making, organizational/OR exp planning techniques/OR exp Health Services Needs and Demand/OR exp healthcare disparities/
6.	(exp Australia/OR Australia$.mp) AND (exp attitude/OR attitude$.mp OR belief$.mp OR knowledge.mp or “unmet need$”.mp)
7.	4 OR 5 OR 6
8.	3 AND 7 (limit publication date to 2001-current)

Only results relating to palliative care models are reported in this paper.

### Study selection

Articles returned from searches of electronic databases were imported into Endnote (version X4) and coded by a single researcher against inclusion criteria for evaluative studies using a standardised proforma.

### Data collection process and data items

Given the rapid nature of our review, we extracted detailed data only from those original studies not contributing to the findings of an included systematic review and data extraction was undertaken by one researcher only. Data on each model of palliative care were extracted using an electronic proforma according to a recently published disease management taxonomy which considered: patient population, intervention recipient; intervention content, delivery personnel, method of communication, intensity and complexity, environment and clinical outcomes [17]. Variables relating to study design, comparator, outcomes and findings were also extracted. Study authors were contacted via email to ask for more information as required.

### Assessment of bias

Systematic reviews were quality rated by a single reviewer using the AMSTAR checklist [18]. Any RCTs we identified that were not included in one or more systematic reviews were rated for quality by a single reviewer using criteria set by the US Agency for Healthcare Research and Quality (AHRQ) [19].

### Synthesis

Models of care were classified according to definitions provided by a range of sources identified by Medline and Google searches; wherever possible, definitions were taken from Australian sources to ensure relevance to the Australian healthcare system [20-33]. Classification was carried out by one reviewer, seeking input from the team as necessary where classification was not straight-forward. Meta-analysis was planned where two or more studies evaluating models of care met criteria set out in the Cochrane Handbook of Systematic Reviews [19]. Where meta-analysis was not possible, synthesis took a narrative approach using techniques described by Popay and colleagues, namely: tabulation, textual descriptions, grouping and clustering, transformation of data to construct a common rubric, vote counting, and translation of data through thematic and content analysis [34-36]. Initial synthesis was undertaken by one author, with each allocated to consider findings in a particular settings (community, hospital, aged care, paediatric and regional/rural). Iterative discussion was used to distil models and elements thereof. In the absence of studies directly comparing different models of palliative care, inference was made from results comparing models with usual care as to which had most evidence for efficacy and cost-effectiveness. No formal methods were used to examine bias across studies.

## Results

Table 2 includes definitions of models of palliative care identified in the literature.

**Table 2:** Definitions of various models or components thereof for palliative care delivery found in the literature

**Model/component**	**Definition(s)**
Case management	Case management is a collaborative process of assessment, planning, facilitation and advocacy for options and services to meet an individual’s holistic needs through communication and available resources to promote quality cost effective outcomes. The definition of case management notes the focus upon the meeting of a client’s health needs. Case management can be placed within a social model of health, within which improvement in health and well-being are achieved by directing efforts towards addressing the social and environmental determinants of health, in tandem with biological and medical factors [31].
Consultation model	An approach to care by which specialist advice is provided on assessment and treatment of symptoms, communication about goals of care and support for complex medical decision-making, provision of practical and psychosocial support, care coordination and continuity, and bereavement services when appropriate [37]. Advice is provided without necessarily assuming primary responsibility for care, although there is negotiation of the level of palliative care involvement.
Health or clinical networks	Health networks are formed when three or more health care agencies (services, organisations or health districts) formally come together to better meet the needs of patients in their service area. These agencies often include hospitals, community health centres, critical access hospitals, physician practices, mental health providers, rural health clinics and other for-profit and not-for-profit health care organizations. These health or clinical networks work to increase access to quality healthcare for local patients and streamline the cost of that care, as well [38].
Integrated care	Integrated care is a concept bringing together inputs, delivery, management and organisation of services relating to diagnosis, treatment, care, rehabilitation and health promotion. Integration is a means to improve the services in relation to access, quality, user selection and offering care [39].
Liaison model	The liaison model combines the education of patients after discharge with educational outreach and clinical support for primary care clinicians. This model may be particularly appropriate in deprived areas, where general practices vary in their capacity to manage chronic illness [40].
Managed clinical networks (MCNs)	Clinical networks are linked groups of health professionals and organisations from primary, secondary, and tertiary care working in a coordinated manner, unconstrained by existing professional and [organisational] boundaries to ensure equitable provision of high quality effective services [21]. MCNs address many of the problems that have been identified in the traditional delivery of health services, including: poor coordination and collaboration between health services; changing roles for health professionals; and the need for greater efficiencies, improved access, more equitable service provision, better use of limited resources and quality patient-centred care. More specifically, MCNs aim to develop locally delivered, quality assured care, through the managed integration of, and cooperation between, formerly separate clinical services. Their major focus is on actively involving patients in service design and building seamless services around the patient’s journey to ensure the best treatment gets to the right patient, at the right time, in the most appropriate place and is delivered by the most qualified and skilled professional with the greatest resources [22].
Pop-up model	Often palliative needs in rural areas may be intermittent or needs specific. Developing a permanent infrastructure would not be appropriate in these circumstances. Looking at available local resources and gaps would provide a basis for developing a ‘pop-up’ palliative service model that optimises how local resources and services can be used to respond to a specific palliative need [24].
Shared care model	A review [28] suggests that three definitions of shared care have been offered:
	1. An approach to care which uses the skills and knowledge of a range of health professionals who share joint responsibility in relation to an individual’s care. This also implies monitoring and exchanging patient data and sharing skills and knowledge between disciplines.
	2. A narrower approach concerned with joint participation of general practitioners and specialists in the planned delivery of care for patients with a chronic condition, informed by an enhanced information exchange, over and above the routine discharge and referral letters.
	3. Especially in mental health, shared care can be divided into systematic cooperation about how systems agree to work together and operational cooperation at local levels between different groups of clinicians.

A total of 1,959 articles returned from electronic databases were screened for inclusion as evaluative studies, of which 23 reported systematic reviews, 9 RCTs and 34 non-randomised comparative studies. Systematic reviews included an average of 18 studies (range 2–43) and varied as to whether they defined their focus by setting (day care [41,42], hospital [43], hospice [44], community [45-49], aged care [50], across settings [51-56]), patient group (transitioning to adult [57], HIV/AIDS [58], dementia [59]) or type of intervention (telehealth [60], caregiver-focused [61], case conferencing [62], UK Gold Standards Framework [63]). Only two of these systematic reviews limited inclusion criteria to RCTs [55,56], although all but three included RCTs alongside other designs. In total, the reviews included 126 RCTs, 29 of which were included in more than one review. Of the 9 RCTs we identified that had not been included in a review, three tested models using case management [64-67] and five SPC consultation [68-72].

**Figure 1:**
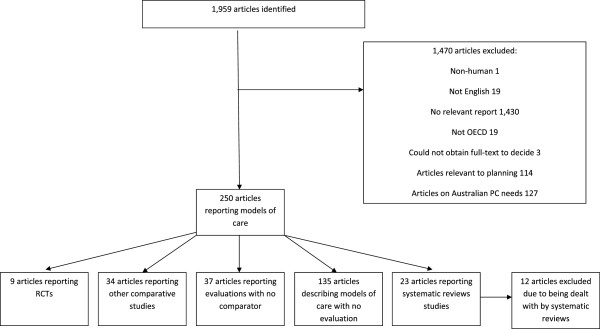
Flowchart for inclusion of articles reporting evaluative studies identified through searches of electronic databases.

See Figure 1 for a flowchart of inclusion/exclusion of peer-reviewed articles and Tables 3 and 4 for details of systematic reviews and RCTs respectively.

**Table 3:** Systematic reviews evaluating the efficacy of palliative models of care

**Review**	**Review question**	**Studies**	**Participants**	**Setting**	**Interventions**	Quality *	**Synthesis**	**Summary of results**
[44]	To identify the current evidence on (1) the effectiveness, including cost-effectiveness, of hospices, and hospice care in a patient’s home and in nursing homes and (2) the experiences of those who use and of those who provide such services	Search date: 2003-2009	People dying from any LLI or their family	Inpatient, RAC, community	EOL care service provided by multidisciplinary team not part of general healthcare	Medium	Narrative	Hospice services are highly valued by patients and their families, reduce general health service use and costs, and increase the likelihood of effective pain management and of death not occurring in hospital
		Designs: comparative and qualitative studies						
		Number of studies: 18 comparative, 4 qualitative						
[41]	To inform future practice, research and policy in specialist palliative day-care by systematically reviewing the evidence for how the structure and process of this form of care relate to outcomes for adults with cancer	Search date: 1872-2003	Patients with cancer	Day care	Palliative day care	Low	Narrative	There is evidence for high satisfaction among patients selected into day-care, but not yet sufficient to judge whether this improves symptom control or QOL.
		Designs: Any, qualitative or quantitative						
		Number of studies: 12						
[57]	To evaluate the evidence on the transition process from child to adult services for young people with palliative care needs	Search date: 1990-2008	Children and young people with LLI	Transition from paediatric to adult services, any setting	Interventions aimed at easing transition	Medium	Narrative	Post-transition patients with cystic fibrosis (and in one study) parents described transition positively
		Designs: any						
		Number of studies: 92, of which only 2 were evaluative						
[45]	To identify the models of inter-professional working that provide the strongest evidence base for practice with community dwelling older people	Search date: 1990:2010	Older (age 65+) people	Community	Inter-professional case management, collaboration or integrated team models	Medium	Narrative	Weak evidence of effectiveness and cost-effectiveness, although well-integrated and shared care models improved processes of care and have the potential to reduce hospital or nursing/care home use.
		Designs: RCTs						
		Number of studies: 41						
[51]	To assess whether there was an effect of palliative care teams	Search date: not given	Patients receiving palliative care and their families	Inpatient or community	Multi-disciplinary teams including staff trained to some extent in palliative care	Medium	Narrative and meta-analysis using effect sizes weighted by the square root of sample sizes	Most evidence is available for home care services (improved satisfaction and pain and symptom control with lower costs), with a smaller number of studies of inpatient hospice or palliative care (similar or greater satisfaction, particularly for carers and similar or improved symptom control, quality of life equivocal) and a small number of poor quality studies considering hospital support, although it does seem that these services reduce time in hospital.
		Designs: not defined						
		Number of studies: 43						
[52]	To identify studies that compare specialised palliative care models between them assessing their effectiveness or cost-effectiveness	Search date: 2003-2006	Terminally ill patients	Inpatient, community	Four hospice-based models (large free-standing hospice, hospital-based hospice, home-care hospice, telemedicine based hospice), one palliative care unit based at a general hospital and two models of referring specialists at hospital (a full service and a service limited to telephonic support to the staff caring the patient)	Medium	Narrative	No differences were found in control of symptoms, QOL, emotional support or satisfaction between a broad service provided by a team of referring specialists at hospital and telephonic support between specialised PCT and the staff caring for the patient. No differences in effectiveness was found between hospital-based hospices and home-based hospice.
		Designs: Comparative						
		Number of studies: 4						
[50]	To determine effectiveness of multi-component palliative care service delivery interventions for residents of care homes for older people.	Search date: to Feb 2010	Elderly people in aged care	RAC	Multi-component palliative care in RAC, including referral to external services or staff training	Good	Narrative	One study reported higher satisfaction with care and the other found lower observed discomfort in residents with end-stage dementia. Two studies reported higher referral to hospice services in their intervention group, one found fewer hospital admissions and days in hospital, the other found an increase in do-not-resuscitate orders and documented advance care plan discussions.
		Designs: Comparative						
		Number of studies: 3						
[46]	Not stated	Search date: 1975-2001	Family members or carers of people with cancer or other advanced disease	Community	Home nursing care, respite services, social networks and activity enhancement, problem solving and education, and group work	Medium	Narrative	The current evidence contributes more to understanding feasibility and acceptability than to effectiveness.
		Designs: Any evaluative						
		Number of studies: 6						
[47]	To update 2003 review to determine the effectiveness of subsequently published intervention studies targeting informal caregivers needs in cancer/advanced disease	Search date: 2001-2010	Family members or carers of people with cancer or other advanced disease	Community	One-to-one psychological models, Psychological interventions for patient/carer dyads, Palliative care/hospice interventions, Information and training interventions, Respite interventions, Group interventions, Physical interventions	Medium	Narrative	Of 6 studies evaluating palliative care/hospice interventions, one pre/post study found reduced family anxiety about caring at home but increased wakening and poorer physical health, one cross-sectional survey found high satisfaction; the others found no effect. The one study evaluating respite found caregivers to report satisfaction after implementation.
		Designs: Any evaluative						
		Number of studies: 33						
[58]	To systematically review the evidence base for the effectiveness of palliative care in improving patient outcomes in HIV/AIDS	Search date: 1980-2003	Patients with HIV requiring symptom control, psychosocial support or terminal care	Inpatient, hospice, community, long-term care	Multidisciplinary advice and support, terminal care, domestic support, care monitoring and planning,	Medium	Narrative	Home palliative care and inpatient hospice care significantly improve patient outcomes in the domains of pain and symptom control, anxiety, insight, and spiritual wellbeing.
		Designs:						
		Comparative Number of studies: 22						
[43]	To determine whether hospital-based palliative care teams improve the process or outcomes of care for patients and families at the end of life	Search date: 1977-1999	Patients with a progressive life-threatening illness, and their family, carers, or close friends.	Hospital	Palliative care teams working in hospitals, defined as two or more healthcare workers, at least one of whom had specialist training or worked principally in palliative care. Included interventions with a hospital/support team component within a broader intervention	Good	Narrative and meta-analysis using effect sizes	All studies except one indicated a small positive effect of the hospital team, including improved symptoms, fewer hospital days and better satisfaction, as well as improvements on process measures such as increased referrals, change in prescribing practices.
		Designs: Comparative						
		Number of studies: 13						
[53]	To determine whether specialist palliative care teams achieve their aims and improve outcomes for patients with advanced cancer and their caregivers, in terms of improving symptoms and quality of life and/or reducing the emotional concerns of family caregivers	Search date: 2000-2009	Patients with advanced cancer and their caregivers	Inpatient, community, outpatient, day care	Specialist palliative care offered by professionals specifically trained in palliative and hospice care	Medium	Narrative	The evidence (moderate and low) supports SPCTs working in home, hospitals, and inpatient units as a means to improve outcomes for cancer patients, such as pain, symptom control, and satisfaction, and in improving care more widely, including reducing hospital admissions. The benefit is demonstrated quantitatively.
		Designs: Comparative Number of studies: 40						
								Studies indicated either benefit in favour of a palliative care team or no difference. Some studies suggested lower costs.
								Quality of life, when measured, less often was different between groups and tended to deteriorate over time.
[60]	To explore the use of telehealth in relation to palliative care in the UK	Search date: 1999-2011	Adults, relatives, or carers with palliative care needs or health professionals in the UK	Inpatient, community, outpatient, RAC	Telehealth	Low	Narrative	Advantages of telehealth include improved access to health professionals and decreased time loss and costs for patients, optimized time use and increased productivity for health professionals, and improved service efficiency for providers. On the negative side, the service becomes depersonalized for both patients and clinicians, confidentiality issues may arise, and potential legal implications for health professionals, including clinical risk management, may be a concern. Clinical staff may be required to perform additional research tasks such as data collection, which might not be considered the main objective when they must compete with the pressures of providing a service. Nonetheless, it appears
		Designs: Any						
		Number of studies: 27						
								to be both feasible and practical to make greater use of telehealth initiatives in order to provide a more equitable palliative care service that is meant not to replace but to enhance the traditional model.
[61]	To provide a comprehensive literature review and critical appraisal of intervention studies with family caregivers of loved ones on hospice	Search date: 1983-2008	Family caregivers of patients receiving palliative care at home in the US	Community	Videophone support, emotional support, self-care/stress reduction, massage	Low	Narrative	Generally positive findings but limited by methodological weakness and mixed results.
		Designs: Any evaluative						
		Number of studies: 5						
[54]	To assess evidence about interventions to improve palliative and end-of-life care	Search date: 1990-2005	People with terminal illness (for example, advanced cancer) and chronic, eventually fatal illness with ambiguous prognosis (for example, advanced dementia)	Any	Case management, coordinated supportive cancer care, nurse care management, in-home support, interventions targeting management and informational and relational aspects of continuity	Medium	Narrative	Moderate evidence supports multidisciplinary interventions that target continuity to affect utilization outcomes. Evidence is strong for reducing readmissions in heart failure, but insufficient evidence was available for other conditions. Successful interventions involved multidisciplinary teaming, addressed patient needs across settings and over time, and facilitated communication by personal and technological means.
		Designs: SRs and any evaluative						
		Number of studies: 9 SRs and 12 studies focused on continuity						
[48]	To establish whether community SPCS offering home nursing increase rates of home death compared with other models	Search date: Earliest records-2011	People with LLI receiving EOL care	Community	Practical nursing support with or without domestic support, education, transition support, tele-support	COI declared	Meta-analysis using OR	Meta-analysis indicated a significant effect on home deaths for SPCS with home nursing versus other care; however, the only two RCTs found no effect. Symptom management or QOL was not compromised and costs were not higher in any study that measured these.
		Designs: Comparative						
		Number of studies:9						
[62]	Does case conferencing improve care planning in palliative patients?	Search date: 1990-2005	Palliative population	Community, inpatient, RAC	Case conferencing between GPs and other healthcare professionals and families	Medium	Narrative	Case conferences were generally acceptable to GPs. Participant perceived benefits included: improved communications between participants; increased GP knowledge about the patient’s illness; interactive discussions with other healthcare professionals as a result of the face-to-face communication; improved inter-professional respect particularly as GPs often did not have a good idea of the roles played by other health providers; a learning opportunity for all participants; a mechanism for de-briefing, particularly when dealing with particularly difficult patients; reduced professional isolation; increased team building and promotion of a team approach to caring for terminally ill patients. Patient outcomes included: assisting in discharge from hospital; improved practice; reduced inappropriate medications, including identification of medication-related problems; increase patient and carer awareness of services; identification and resolution of problems; reduced primary care visits; maintenance of function and independence; increased use of services. No effect observed on quality of life or survival; effect seemed to be limited to outcomes the care teams had direct influence on.
		Designs: Any evaluative						
		Number of studies: 20						
[59]	To test the efficacy of a palliative care model in patients with dementia	Search date: 1966-2003	People with advanced dementia	RAC, acute hospital	Dementia Special Care Unit (DSCU), palliative care plans aimed at maximising comfort and minimising invasive or aggressive treatment (including hospitalisation)	Medium	Narrative	Patients in the DSCU had lower discomfort and fewer transfers to acute medical setting but higher mortality; the study in the acute hospital setting found no effect on length of hospital stay or reduction in painful interventions.
		Designs: Any evaluative						
		Number of studies: 2						
[49]	To determine if providing home-based end of life care reduces the likelihood of dying in hospital and what effect this has on patients’ symptoms, quality of life, health service costs and care givers compared with inpatient hospital or hospice care.	Search date: 1950-2009	Adults receiving terminal care at home who would otherwise require hospital or hospice inpatient care.	Community	EOL care at home providing active treatment for continuous periods of time by multidisciplinary healthcare professionals	Medium	Meta-analysis using risk ratio	Those receiving home-based end of life care were statistically significantly more likely to die at home. There was some evidence of increased patient satisfaction with home-based end of life care, and little evidence of the impact this form of care has on caregivers. No statistically significant differences were found for functional status, psychological well-being or cognitive status.
		Designs: Comparative						
		Number of studies:4						
[42]	To determine whether the provision of palliative day care services (PDS) have a measurable effect on attendees’ wellbeing	Search date: 1978-2009	Patients with LLI	Day care	Holistic, individualized palliative care, including medical and nursing care, allied health and complementary therapies, social support,	Medium		Little evidence of impact on QOL but people report that attending PDS is a valuable experience that allows them to engage with others and to be supported in a restorative environment.
		Designs: Any evaluative						
		Number of studies:35						
[63]	To review the impact of the Gold Standards Framework (GSF) since its introduction to the UK in 2001	Search date: NR-2008	People receiving EOL care	Primary care	Toolkit to improve the quality, coordination and organisation of EOL care	Medium	Narrative	Evaluation to date has focused on the GSF’s impact on care processes rather than outcomes. The GSF has proven acceptability and can influence multidisciplinary collaboration, communication, assessment and care planning
		Designs: Any						
		Number of studies:27						
[55]	To identify and analyse all published RCTs that focus on the organization of EOL care provided to persons who are terminally ill, near death, or dying	Search date: NR	People who are terminally ill, near death or dying	Inpatient, community	Multidisciplinary care, staff training	Low	Narrative	Community or home-based EOL care compares favourably with more traditional or conventional hospital-based and episodic medical care in improving symptoms and in the opinions of patients and caregivers
		Designs: RCTs						
		Number of studies:23						
[56]	To systematically review the evidence for effectiveness of specialized palliativecare	Search date: earliest records-2007	Terminally ill	Community, inpatients, outpatients	Multidisciplinary care and support, education, caregiver support, coordination,	Medium	Narrative	Evidence was most consistent for effectiveness of SPC in improvement of family satisfaction with care (7 of 10 studies). Only 4 of 13 studies assessing QOL and 1 of 14 assessing symptoms showed a significant benefit of the intervention; however, most studies lacked statistical power. There was evidence of significant cost savings in only 1 of 7 studies that assessed this outcome.
		Designs: RCTs						
		Number of studies: 22						

COI: conflict of interest; EOL= end of life; LLI = life limiting illness; RAC = residential aged care; SR = systematic review. *Quality has been rated using AMSTAR as follows: Good quality = 8–11; medium quality = 4–7; low quality = below 4 [18].

**Table 4:** Randomised controlled trials (RCTs) comparing models of care to ‘usual care’ and reported in the peer-reviewed literature

**Ref**	**Model of care**	**Setting/**	**Referral/**	**Delivery personnel**	**Communication/**	**Intensity/**	**Comparator**	**Outcomes**	**Findings**	Quality*
		**population**	**access**		**coordination**	**complexity**				
[64]	Case management	Community-dwelling ‘seriously chronically ill’ (<2 year life expectancy)with COPD or CHF (N=192)	Patients receiving treatment from one of multiple managed care organizations	Nurse case-managers, supported by medical director, social worker and pastoral counsellor	Primary care physician, health plan case manager and community agencies	NR	Usual care, including telephone-based medical and disease- oriented case management	Self-management, preparation for EOL, symptoms, QOL, medical service utilisation	IG reported lower symptom distress, greater vitality, better physical functioning and higher self-rated health. ED utilisation was equivalent across groups	Poor
[65,66]	Case management	Rural community-dwelling patients newly diagnosed with advanced cancer (N=322)	Patients identified by the VA Medical Centre’s tumour board	PC advanced practice nurses, supported by PC physician, psychologists, and ‘other team members’	Referral to medical teams and community resources as required	4 face-to-face sessions with monthly telephone follow-up and group shared medical meetings	Usual care at VA Medical Centre	QOL, symptoms, depression, days in hospital, ED visits	IG higher scores for QOL and mood, but did not have improvements in symptom intensity scores or reduced days in the hospital or ICU or ED visits.	Good
[68]	Consultation	ICU inpatients with a terminal or preterminal condition (N=20)	Patients identified by intensivist indicating that (s)he believed treatment should not be escalated or should be withdrawn	PC physician, registrar, resident and clinical nurse consultant	None indicated	Daily ward rounds	Usual ICU care	ICU and hospital length of stay and satisfaction with quality of care of families, intensivists, and bedside nursing staff, ICU and hospital mortality, the number of medical teams caring or consulting for the patient	No statistically significant differences	Poor
[69]	Consultation	Hospital inpatients with LLI	Referrals received from all medical services and inpatient units	PC physician and nurse, hospital social worker and chaplain	Liaised with hospital subspecialists, attended discharge meetings, electronic discharge information sent to GP	NR	Usual inpatient care	Symptom control, levels of emotional and spiritual support, patient satisfaction, total health services costs, survival, number of advance directives at discharge, and hospice utilisation	IG had fewer ICU admissions, lower 6-month net cost savings, and longer median hospice stays. There were no differences in survival or symptom control.	Good
[67]	Case management	Oncology inpatients and outpatients referred to PC service (N=159) and their caregivers	Referred by oncology inpatient or outpatient services	SPC service NOS, GP	Follow-up communication in both arms via faxed or posted letters, and telephone communication between family physician and specialist, or domiciliary nurses present at specialist team meetings acting as an intermediary	Single case conference via telephone and follow-up as required	Usual oncology inpatient or outpatient care	QOL, caregiver burden	No significant differences in magnitude of change in QOL from baseline but IG showed better maintenance of some physical and mental health measures of QoL in the 35 days before death	Poor
[70]	Consultation	Outpatients with New York Heart Association functional classes III and IV CHF (N=13)	NR	SPC NOS	NR	Initial consultation + monthly for 5 months	Usual cardiology care	Anxiety, depression and QOL	Low recruitment and attrition precluded analysis	Poor
[71]	Consultation	Acute care inpatients with advanced dementia (N=32) and their caregivers	Recruited from acute medical wards	SPC NOS	Copies of ACPs were placed in the medical notes and sent to GPs and RAC (where relevant)	Up to 4 consultations	Usual inpatient care	Caregiver distress, decision satisfaction, QOL and (if the patient died) satisfaction with EOL care	Attrition precluded analysis	Poor
[72]	Consultation	Oncology outpatients with newly diagnosed metastatic non–small-cell lung cancer (N=151)	Recruited from oncology outpatients	PC palliative care physicians and advanced-practice nurses	Care coordination NOS	Average number of 4 SPC visits	Usual oncologic care	Anxiety, depression, QOL, survival, health service use	IG had higher QOL, lower depression and longer survival despite less aggressive EOL care	Good

ACP = advance care plan, COPD = chronic obstructive pulmonary disease, CHF = chronic heart failure, ED = emergency department, EOL = end of life, IG = intervention group, ICU = intensive care unit, LLI = life-limiting illness, PC = palliative care, (N) = total sample size at baseline, NOS = not otherwise specified, NR = not reported, QOL = quality of life, RAC = residential aged care, SPC = specialist palliative care; *Quality was rated as ‘good’, ‘fair’ or ‘poor’ according to criteria for internal validity set by the US Agency for Healthcare Research and Quality [73].

In keeping with international policy, this review found a high level of interest in addressing the palliative care needs of populations beyond people with cancer to those with illnesses such as chronic heart failure [74], end-stage kidney disease [75,76], chronic obstructive pulmonary disease [77] and dementia [78]. Research has highlighted the importance of better identifying the palliative phase of these conditions in order to appropriately time advance care planning, access to symptom management and provision of support to patients and their families. Many studies included patients with a range of diagnoses and did not distinguish care or effectiveness by disease group.

Variation in the content of models, contexts in which these were implemented and lack of detailed reporting meant that no two studies met the requirements for meta-analysis that had not previously been reported in a published review. Heterogeneity in the ways models were configured and described led to a focus on the attributes of effective palliative care and service elements effective at delivering these as the most meaningful unit of analysis, rather than models of care *per se*.

### Attributes of effective palliative care

Table 5 contains a summary of the attributes of palliative care provision recommended by English-language national policies from OECD countries.

**Table 5:** Attributes of models for palliative care recommended by national policy documents from OECD countries available in English

**Country**	**Attributes of palliative care service delivery recommended by national policy**
Australia [23]	• Provide enhanced, coordinated support for carers, volunteers, communities of carers and carer respite
	• Provide coordinated, flexible local care delivery for people at the end of life regardless of where they live and address any barriers
	• Further improve the skill and confidence of the generalist workforce
	• Enhance online palliative care support to ensure adequate numbers of skilled palliative care specialist providers across all disciplines
	• Include end of life and palliative care competencies in all care worker training packages
	• Enhance and legitimise the role of specialist consultancy services in providing direct clinical advice, education and training, advocacy for end of life issues and training places
	• Record and track advance care planning within electronic health records
	• Develop sustainable models of quality palliative care in the private sector
	• Develop the role of the general practitioner in palliative care
	• Undertake further research and ongoing monitoring of the relative cost of care
Canada [79]	• Availability and access to services
	• Education for healthcare providers
	• Ethical, cultural and spiritual considerations
	• Public education and awareness
	• Support for family, caregiver and significant others
Ireland [80]	• Provision of physical, psychological, social and spiritual support, with a mix of skills, delivered through a multi-professional, collaborative team approach
	• Patients and families are supported and involved in management plans
	• Patients are encouraged to express their preference about where they wish to be cared for and where they wish to die
	• Carers and families are supported through the illness into bereavement
	• The overall whole time equivalent (WTE) SPC nurse to bed ratio should not be less than 1:1
	• In each day care centre, there should be a minimum of one WTE SPC nurse to every 7 daily attendees.
	• There should be a minimum of one WTE specialist palliative care nurse per 150 beds in each acute general hospital
	• There should be a minimum of one WTE specialist palliative care nurse in the community per 25,000 populations.
	• There should be at least one WTE physiotherapist per 10 beds in the specialist palliative care inpatient unit, with a minimum of one physiotherapist in each unit
	• There should be a minimum of one WTE community physiotherapist specialising in palliative care per 125,000 population. This post should be based in the specialist palliative care unit
	• There should be at least one WTE occupational therapist per 10 beds in the specialist palliative care inpatient unit, with a minimum of one occupational therapist in each unit.
	• There should be a minimum of one WTE community occupational therapist specialising in palliative care per 125,000 populations. This post should be based in the specialist palliative care unit
	• There should be at least one WTE social worker employed per 10 beds in the specialist palliative care unit, with a minimum of one social worker in each unit
	• There should be a minimum of one WTE community social worker specialising in palliative care per 125,000 population. This post should be based in the specialist palliative care unit
	• Specialist palliative care services in all other settings, including general hospitals and the community, should be based in or have formal links with the specialist palliative care unit
	• All specialist palliative care units should provide day care facilities for patients and carers
	• Appropriate transport should be provided for patients to and from the centre
	• There should be one point of entry to hospital services for palliative care patients, and subsequent referrals should be speedily organised
	• In Accident and Emergency, the patient’s condition should be rapidly assessed, and the patient should be referred to the appropriate team without delay
	• The specialist palliative care team in the community should be an inter-disciplinary consultant-led team
	• The specialist palliative care team should be based in, and led by, the specialist palliative care unit in the area
	• Specialist palliative care nurses should provide a seven-day service to patients in the community
	• Arrangements should be made for the transport of patients receiving palliative care to different care settings, when required
	• Bereavement support should begin early in the disease process, long before the death of the patient.
	• Multidisciplinary assessment to ensure that all needs are identified early and individualised plan is established
	• Allocate a care coordinator to each dying person
	• Provide access to clinical care for each dying person (medical services, respite care, counselling, etc.)
New Zealand [81]	• Provide access to support services for dying patients and their families
	• Ensure dying people and their families have access to essential palliative care (initial and specialized palliative acre)- at least one local palliative care service in each district health board
	• Provide induction and ongoing training for volunteers in the community assisting in palliative care
	• Provide flexible palliative care to meet varying and specific needs
	• Inform the public about PCS.

Our review of research evidence found that few studies have been conducted across care settings, with most focusing on the provision of palliative care either in the community, acute care or aged care settings.

#### ***Attributes of home-based models of palliative care***

Most commonly, models of palliative care have been aimed at supporting home-based end of life care, optimising use of SPC expertise, avoiding futile treatments and providing support for family-care givers and community health professionals [44-49,51-53,55,56,58,61]. The most important characteristics of home-based models of care have been documented as those that support communication and coordination, engage and enable skill enhancement both for the primary palliative care team (including general practitioners [GPs]) and informal caregivers/patients, and clarify goals of care through advance care planning.

#### ***Attributes of acute care models of palliative care***

Models of palliative care adopted in the acute care sector largely consist of specialist consultative services, in-patient palliative units/beds or nurse practitioner models [82,83]. In a landmark study from the US, SPC consultation was found to improve not only quality of life but also surival for patients with advanced lung cancer [72]. Specialist consultative service models have tended to focus on: 1) discussions about prognosis and goals of care; 2) pursuing documentation of advance directives; 3) discussion about foregoing specific treatments and/or diagnostic interventions; 4) family and patient support; 5) discharge planning; and 6) symptom management [84]. Consultative services provided by hospital palliative care teams have been shown to improve symptom control and quality of life, alleviate emotional burden and improve caregiver and patient satisfaction [85,86]. In addition they have resulted in hospital cost saving [87,88]. Currently, SPC in the US acute care setting is more likely to be available in larger hospitals, academic medical centres, not-for-profit hospitals, and Veterans Affairs (VA) hospitals compared to others [89]. Dedicated palliative care units have been established but struggle to meet increasing demands.

The increasing pressure on emergency departments and recognition of their role in end-of-life care highlight the dearth of community based services and failure of advance care planning [90-92]. Commonly, emergency presentations result from inadequate symptom control in the community and/or absence of adequate care givers [93-95]. In some countries, financial issues also act as an incentive for patients to access treatment through the emergency department in preference to other services [96]. A particular issue is the uncertainty that emergency department health professionals face when forced to make decisions in the absence of a detailed case history and advance care plans [92]. Studies have identified the capacity of coordinated models of care to decrease unnecessary emergency department usage and inappropriate admission, especially to intensive care [97,98].

#### ***Attributes of residential aged care models of palliative care***

A setting that has proven especially challenging to improvements in quality of end of life care is residential aged care [99]. Older people in aged care are less likely to be referred to SPC services for consultation or ongoing management and more likely to have poor symptom control, unnecessary hospitalisations, sub-optimal communication, inadequate advance care planning and families who are dissatisfied with end of life care [50]. A recent Cochrane Review [50] examining multi-component palliative care interventions for older people in nursing homes identified three studies [100-102] graded as ‘poor quality’ that provided weak evidence for the following model of palliative care elements: i) *communication* - identifying residents who would benefit from an SPC referral and negotiating this with their doctor and family [100]; ii) *development of palliative care leadership teams*, technical assistance meetings for team members, education in palliative care for all staff, plus feedback on performance [102]; and iii) *targeted symptom control strategies* to improve discomfort [101]. Systematic reviews on the efficacy of palliative care in dementia have identified a very limited evidence-base with which to develop appropriate interventions or services [54,59].

#### ***Attributes of care required during transitions***

Models of care are faced with special challenges during transitions between care settings (community, aged care and hospital) where support is needed to avoid patients ‘falling through the cracks’ [103] and/or when a rapid response is required in the context of quickly changing clinical status or patient preferences for place of care (e.g. wishing to return home while still possible) [104]. As patients and caregivers may lack knowledge of what services are available and how to access them [105], navigating the transition from inpatient to community based care requires intensive effort and coordination to put management plans and caregiver support in place. The importance of supporting transitions is especially underscored in advanced dementia where, unless a care plan is in place, health professionals in acute care may lack awareness that a palliative approach is appropriate and initiate treatments inappropriately aimed at prolonging life with negative effects on quality of life [106,107]. Transitional care between paediatric and adult palliative care services is also a focal point requiring intensive support [57].

### Elements of effective models of palliative care

This review identified a number of dynamic elements that have been integrated into palliative care models in a range of care settings to enable access to appropriate services, improve communication and coordination between providers, enhance palliative care skills of non-specialist and informal carers, and inrease capacity to respond rapidly to individual patient needs and preferences as these change over time.

#### ***Case management***

Case management is a recurring feature of many successful models [43-45,48,49,51,53-56,58,62,64-69] that seeks to assess and meet the full range of each individual’s palliative care and other needs, including those relating to activities of daily living (e.g. house-work) and social wellbeing. As a result, case management frequently requires coordination of services beyond the healthcare sector, including social services and pastoral care. Case management is informed by the principles of patient-centred care [108]; as such, patients and families themselves often play an active role in determining which services they receive.

#### ***Shared care***

Whilst defintions of shared care have varied (Table 2), it has been frequently reported as an element of effective palliative care delivery, utilised by a number of different models [109]. Characteristics of shared care seem to have commonly included: an identifiable lead clinician working together with health professionals from other disciplines, a focus on communication and coordination, and a rapid needs-based response and navigational strategies.

A model of care that incorporates case management and shared care and has been recommended by policy in Australia in the absence of evaluation data is the ‘pop up’ model. This model was originally developed to extend palliative care to rural/remote adult services and has since been recommended for paediatric palliative care [110]. The model develops a rapid-response team around the patient and their family drawn from primary, community-based and SPC services as required to address each client’s care plan. The model relies on excellent coordination, established networks and a system of triggers for referrals, re-assessments and re-referrals to provide intensive support over brief periods. In the UK, a coordinating role for a similar model has been assigned to paediatric oncology outreach nurse specialists to support children dying from cancer [111,112]. The outreach nurse role is described as ‘empowering the primary healthcare team through advice and direct patient care; providing an interface between primary, secondary, and tertiary care services; and coordinating services’ [111] (p.4474).

#### ***Specialist outreach services***

Internationally, specialist outreach services have been widely adopted to improve care outcomes for underserved populations through the establishment of: i) specialist clinics in urban primary care practices; ii) specialist clinics in rural hospitals where no specialist services exist; and iii) sub-specialist clinics in regional centres [113]. A Cochrane review examined efficacy of specialist outreach services in primary care and rural hospital settings implemented as one element of complex multifaceted interventions involving collaboration with primary care, education or other health services [113]. This review concluded that specialist outreach services can improve health outcomes, ensure delivery of more efficient and consistent evidence-based care, and reduce the use of inpatient services. The additional costs associated with the provision of specialist outreach appear to be balanced by improved health outcomes. None of the studies in the review included comparisons of palliative care specialist outreach services; their widespread use raises a need for evaluation [114].

#### ***Managed clinical networks and/or health networks (clinical networks)***

Across the globe, clinical networks have been integrated into many healthcare systems as part of a wider reform agenda to ensure that underserved populations and those with poorer outcomes have better access to quality, clinically-effective health services [115,116]. Clinical networks facilitate the formal linking of groups of health professionals and organisations from primary, secondary and tertiary care to work in a coordinated manner, unconstrained by existing professional and organisational boundaries [117]. Many of these boundaries are driven by funding models and geographical boundaries. Although conceptually appealing, few empirical studies have been undertaken to evaluate the effectiveness of clinical networks. A literature review identified eight empirical studies, including comparative and observational designs [117]. The review concluded that clinical networks - when formally established, with governance and guidelines in place - facilitated access to care for people in underserved communities.

#### ***Integrated care***

Numerous studies identified the crucial role of integrated care [51-56]. Integration refers to coordination of disparate services centred on the needs of each individual patient and family with the aim of ensuring continuity of care. Integrated care requires that patients and families are involved in informed decision-making and goal setting. It is based on principles of advocacy and respect that provide seamless, continuous care from referral through to bereavement and across organizational boundaries. Positive effects of integrated care in paediatrics have been demonstrated not only for patient and family outcomes, but also on organisational efficiencies and staff satisfaction [57].

Integrated care is especially important when supporting adults or children in the community, the enablement of which is increasingly prioritised by policy in many countries [118,119]. While the role of primary care at the end of life is important everywhere, palliative care support for primary healthcare is most essential in rural and regional areas, where the burden for coordinating and providing medical care falls predominantly on general practitioners (GPs) and nursing care to community nurses [120,121]. Data suggest that in some jurisdictions, including Australia, many GPs want to be involved in palliative care delivery but have decreasing capacity to undertake visits to homes or aged care facilities due to workload, time constraints and inadequate remuneration [7,122-126]. Whilst there are no evidence-based models for palliative care in the primary healthcare setting [127,128], there is emerging evidence that the UK’s Gold Standards Framework (GST) has improved communication, collaboration, assessment and planning since its introduction in 2001 [63]. It should be noted, however, that the UK’s National Health Service has unique drivers not readily transferrable to countries such as Australia with different healthcare funding models and multiple jurisdictions.

#### ***Volunteers***

Use of volunteers may have potential where informal caregivers are lacking; however, appropriate governance models are needed. Volunteer models have been used across a range of palliative care settings but evidence of implementation and evaluation is limited [129-132].

### Cost-effectiveness

Most studies that have examined cost-effectiveness of palliative care services versus usual care have found either no significant difference or palliative care to compare favourably [44,45,48,51,53,56]. However, there remains controversy as to appropriate methods of measuring cost-effectiveness in care for the dying. The limited survival of this patient population proves a challenge for cost-utility methods; most analyses to date have focused on costs alone, with little integration of data on efficacy. Furthermore, relatively little attention has been given to costs incurred by family caregivers who may absorb costs shed by the healthcare system via community care interventions aimed at avoiding hospital admissions. No data were found comparing cost-effectiveness of different models of palliative care beyond usual care.

## Discussion

Like previous systematic reviews in palliative care [133], we found few well-designed RCTs comparing models of palliative care with each another, or even with usual care. Systematic reviews have tended to include service-level interventions defined by setting (e.g. day care [42]) and/or the population served (e.g. people with dementia [59]) rather than by model of care. This consideration led us to redirect our synthesis away from whole models to focus on service elements consistently featured in models found to be effective. Of these elements, case management has been perhaps the most commonly supported [43-45,48,49,51,53-56,58,62,64-69], albeit usually contributing to a complex intervention alongside a number of interacting components, different in each study. These considerations limit our ability to state with confidence that positive outcomes have resulted from case management *per se*.

Our review also identified the role required of political drivers in leveraging health system reform. Case management demands care across jurisdictions and care settings, which is not easy to achieve in a state/federal funding structure of the kind seen in Australia. The influence of local drivers also means that some models of care have been based on geo-political boundaries that may not be readily transferrable to other regions [63,134-136].

Two new systematic reviews published since our search was conducted have provided important data on cost-effectiveness of palliative care. The value of home based palliative care has been demonstrated in a recent meta-analysis which found that receiving home palliative care doubles the odds of dying at home and reduces symptom burden, especially for patients with cancer, without having an adverse impact on caregiver grief [137]. A systematic review using narrative synthesis concluded that palliative care of all kinds was generally found to be cost-effective compared with usual care, usually statistically so [138].

### Limitations and areas for future research

The rapid nature of the current review is associated with a number of methodological limitations [12,13]. Limiting the scope of our search and associated terms is likely to have resulted in relevant references having been missed and increased the risk of publication and country/language biases [139]. Our inclusion criteria and approach to synthesis favoured reviews over original research and relied on a somewhat ‘blunt’ comparison that did not control for overlap between reviews. Limitations in time and resources also required us to forego the level of documentation commonly associated with full systematic reviews. These limitations were moderated somewhat by the use of the online resource ‘CareSearch’ which was designed by experts specifically to identify palliative care evidence [140] and quality assessment involving experts, including the authors of key research [141]. However, the emphasis we placed on models of care relevant to the Australian healthcare system will inevitably limit applicability of findings to some other countries.

As mentioned, the current review was also limited by variations in reporting of service models that precluded comparison and accumulation of evidence for any given model. The term ‘model of care’ was itself used inconsistently and relatively infrequently in the literature; a Medline search using terms for ‘palliative care’ combined with ‘model(s) of care’ returned only 1% of articles returned by searching for palliative care alone. Inconsistency and incompleteness in reporting impairs not only synthesis of research but also replication of successful models in future evaluations and implementation into practice. Researchers are encouraged to follow guidance on key variables to report that would enable greater comparability and support replication and refinement of models in research and practice [142].

The literature’s focus on elements rather than models raises important questions about how these elements might interact to the betterment or detriment of care quality and outcomes. The pop up model is one example of a model of care that has been recommended by policy without evidence for its effectiveness as a whole but rather an assumption that effective elements can be combined to optimise benefit [110]. Future evaluations should use factorial designs and process measures to clarify causal mechanisms between elements and identify influential contextual factors to inform ongoing development and tailoring to local needs and resources [143].

Finally, our review was limited by the problem we encountered in mapping between evidence at the outcome levels of patient (e.g. symptoms), caregiver (e.g. satisfaction), provider (e.g. knowledge of palliative care needs) and service (e.g. hospital days). A recent systematic review identified 15 patient-level domains alone, including quality of life, quality of care, symptoms and problems, performance status, psychological symptoms, decision-making and communication, place of death, stage of disease, mortality and survival, distress and wish to die, spirituality and personality, disease-specific outcomes, clinical features, meaning in life and needs [144]. The plethora of outcomes and associated measures is a recognised barrier to comparability between studies [145-150]. Whilst the WHO palliative care definition provides a framework for evaluating palliative care at the levels of the patient, provider and system, this has not yet been undertaken for any known model of palliative care. There is also a need for comprehensive economic evaluations that include descriptions of patient preferences as well as consideration of costs incurred by family caregivers and sub-group analyses examining the influence of disease and socio-demographic factors [138,151].

## Conclusion

Heterogeneity in definitions and reporting mechanisms limit the focus of conclusions from this rapid review to attributes and elements of successful palliative care services rather than whole models. Best practice palliative care should be accessible to all who need it, tailored to individual patient and family’s palliative care needs in a timely manner, and extend beyond organisational and disciplinary boundaries as required via strategies that support communication and coordination. Population-based models of palliative care should therefore include elements that support case management via integration of SPC with primary and community care services, and enable transitions across settings, including residential aged care.

While palliative care models may have once been relatively homogenous, dynamic models are increasingly required to accommodate rapidly changing population demands and health system structure and drivers. Access to specialist services for rural and regional patients and carers has been identified as especially in need of targeted intervention. The current focus on medical and nursing service delivery should also be broadened to incorporate services addressing social and environmental determinants of health as required.

Increasing complexity in service configuration warrants consideration by future research of the roles played by contextual factors such as funding and policy in order to inform planning at the population level. Research should ideally test the impact of changes over time both within and between regions using standard measures of process and outcomes.

## Abbreviations

GP: General practitioner; SPC: Specialist palliative care.

## Competing interests

The authors declare that they have no competing interests.

## Authors’ contributions

All authors contributed to design and conduct of the review, synthesis and interpretation of results and reporting. All authors read and approved the final manuscript.

## Pre-publication history

The pre-publication history for this paper can be accessed here:

http://www.biomedcentral.com/1472-6963/14/136/prepub
